# Diagnosis and Surgical Management of Congenital Intranasal Teratoma in a Newborn: A Rare Case Report

**DOI:** 10.1155/2018/1403912

**Published:** 2018-04-19

**Authors:** W. X. Yeo, K. K. Tan

**Affiliations:** Department of Otolaryngology, KK Women's and Children's Hospital, Singapore

## Abstract

Teratomas are the most common germ cell tumors of childhood. Head and neck teratomas, however, account for less than five percent of all teratomas. Considered rare at an incidence of 1 in 20,000 to 40,000 live births, they may occur in the cervical region, nasopharynx, brain, orbit, or oropharynx. Teratoma presenting as an isolated intranasal mass is extremely rare. In this report, we describe a case of a mature teratoma arising from the roof of the nasal cavity presenting as an isolated intranasal mass, the first of its kind from our literature review. The tumor was resected endoscopically with no recurrence detected.

## 1. Introduction

Teratomas are the most common germ cell tumors of childhood [1], consisting of tissues from each of the three embryonic germ layers (ectoderm, mesoderm, and endoderm). They are mostly benign [2], although malignant transformation has been infrequently described [3, 4]. Teratomas of the head and neck account for less than five percent of all teratomas [5, 6], occurring in about 1 in 20,000 to 1 in 40,000 live births [7]. They are most commonly found in the cervical region and the nasopharynx [8, 9], areas which may result in respiratory distress or issues. Occurrences have also been described in other head and neck regions such as the brain [2], orbit [10], and oropharynx [11]. Teratoma presenting as an isolated intranasal mass is extremely rare. In this report, we describe a case of a mature teratoma arising from the roof of the nasal cavity presenting as an isolated intranasal mass, the first of its kind from our literature review. The tumor was successfully resected endoscopically.

## 2. Case Report

A full-term newborn boy presented with noisy breathing and mild subcostal retractions at birth. There was no stridor or desaturations on room air. On examination, there were no external nasal deformities or swellings. Bedside nasoendoscopy revealed a soft fleshy mass occupying the left nasal cavity. An attempt to pass a 6-French catheter beyond the mass into the nasopharynx was successful, which suggested that there was no associated choanal atresia. Examination of the right nasal cavity, apart from a septal deviation as a result of mass effect from the left nasal mass, was otherwise unremarkable. The oral cavity and oropharynx were also clear with no evidence of cleft palate.

**Figure 1:**
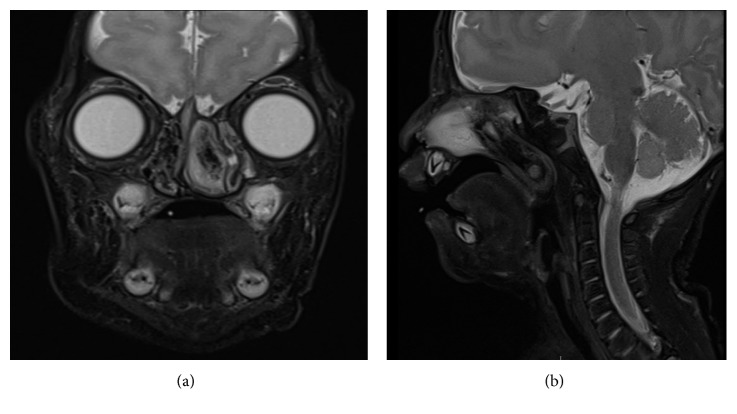
MRI T2-weighted axial (a) and sagittal (b) images depicting a left nasal mass arising from the roof of the nasal cavity and extending to the posterior choana, with no intracranial extension seen.

Computed tomography (CT) and magnetic resonance imaging (MRI) scanning of the nose were performed, which revealed a left nasal mass lesion arising from the roof of the nasal cavity with extension into the posterior choana (Figure 1). There were no underlying bony skull base defects, nor any herniation of intracranial structures to suggest a nasal encephalocele. In view of the presence of an isolated intranasal mass, the differential diagnoses considered at the time of presentation were congenital midline nasal mass (e.g., nasal glioma) and nasal tumor (e.g., nasal chondromesenchymal hamartoma, or nasal teratoma).

**Figure 2:**
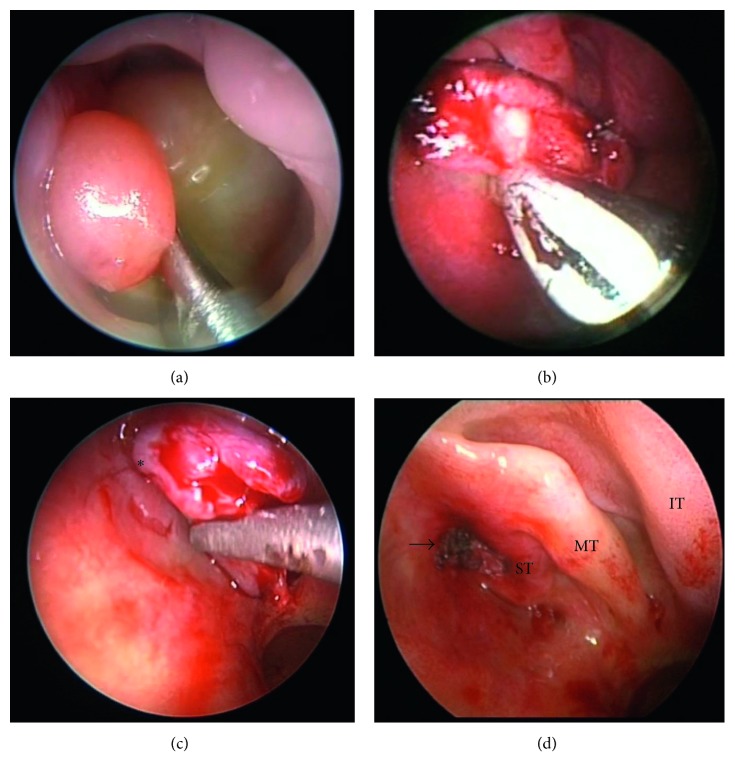
Intraoperative images depicting a large fleshy left nasal mass (a), which was endoscopically debulked with a microdebrider (Skimmer) (b). The stalk of the teratoma (asterisk) was revealed to be arising from the roof of the nasal cavity (c). Postresection image (d) showing the site of tumor origin (arrow) in relation to the turbinates. ST, superior turbinate; MT, middle turbinate; IT, inferior turbinate.

The patient subsequently underwent an examination of the nose and endoscopic resection of the nasal mass under general anesthesia. Intraoperatively, a large left nasal mass was noted (Figure 2(a)), which extended anteriorly to the nasal vestibule and posteriorly to the posterior choana. An incisional biopsy was performed, and intraoperative frozen section revealed a “stratified squamous epithelium, fat, and skeletal muscle lesion—in keeping with a teratoma.” The tumor was gradually debulked and removed endoscopically with a microdebrider (Skimmer®) (Figure 2(b)). The stalk of the tumor was traced to be arising from the roof of the nasal cavity medial to the superior turbinate (Figures 2(c) and 2(d)). Nearing the skull base, the stalk of the tumor was carefully removed with Tru-Cut blades. Any visible remnant tumor tissue was cauterized with suction diathermy to ensure complete removal of the tumor. Care was taken at this point to prevent any breach of the skull base which might have otherwise resulted in a cerebrospinal fluid (CSF) leak.

Postoperatively, the patient had an uneventful recovery and was discharged well on postoperative day 3. Final histology confirmed the diagnosis of a mature teratoma, with presence of tissues from all three germ layers (e.g., stratified squamous epithelium with adnexal structures (ectoderm), skeletal muscle (mesoderm), and ciliated respiratory epithelium (endoderm)). On follow-up at three weeks and three months postresection, there was no evidence of tumor recurrence on examination under general anesthesia (which allowed for optimal examination of the nasal cavity). The patient is currently being followed up every six months.

## 3. Discussion

The nasopharynx is a common site of origin for congenital head and neck teratomas, which is often associated with cleft palate formation [12]. In comparison, congenital teratomas arising from the nasal cavity are much rarer. A handful of cases have been described arising from the nasal septum [13–16], either with or without an associated cleft palate. One case described a congenital teratoma arising from the nasal vestibule [17]. To the best of our knowledge, this is the first report of a congenital teratoma arising from the roof of the nasal cavity.

An intranasal mass in a newborn often triggers the consideration of a congenital midline nasal mass such as a nasal encephalocele or a nasal glioma [18]. This is especially so when the mass is seen arising from the roof of the nasal cavity, as with this case. Preoperative imaging with MRI and CT is therefore essential to assess for any intracranial extensions or bony skull base defects suggestive of a nasal encephalocele [19, 20]. This is because treatment is vastly different and would require a multidisciplinary team involving the expertise of a neurosurgeon [21]. Apart from congenital midline nasal masses, tumors have to be considered as well. Tumors may be benign or malignant [22], of which teratoma is one of the diagnoses.

As this was an isolated intranasal teratoma, resection was performed via an endoscopic approach. A Skimmer microdebrider was chosen for its small size, which permitted the debulking of the teratoma within the confines of a neonatal nasal cavity. Similar successful cases of endoscopic resection have also been previously described for nasopharyngeal [23] and nasal septum teratoma [15], although the choice of surgical approach (open versus endoscopic) is usually dictated by the location and extent of the disease. Other surgical modalities have also been described, including radiofrequency coblation of a nasopharyngeal teratoma [24] and CO_2_-laser resection of an extensive nasopharyngeal and oral teratoma [25].

In general, the long-term prognosis of head and neck teratomas following surgery is excellent [3], as teratomas are mostly benign tumors with low recurrence rates so long as resection is complete. Routine follow-up for recurrence should include physical examination and imaging studies when appropriate [26]. Periodic measurements of serum alpha-fetoprotein (AFP) levels may also be performed, as teratoma recurrences have been detected by elevations of AFP levels after resection [27, 28].

In summary, we report the first case of a congenital teratoma arising from the roof of the nasal cavity that has been successfully resected via an endoscopic approach.
